# Feeding Infants and Toddlers Studies (FITS) Provide Valuable Information for Setting Dietary Guidelines

**DOI:** 10.3390/nu14194073

**Published:** 2022-09-30

**Authors:** Susan J. Whiting, Tolassa W. Ushula

**Affiliations:** 1 College of Pharmacy and Nutrition, University of Saskatchewan, Saskatoon, SK S7N 5E5, Canada; 2 Nutrition and Dietetics Department, Faculty of Public Health, Jimma University, Jimma 378, Ethiopia; 3 School of Public Health, Faculty of Medicine, The University of Queensland, 266 Herston Rd, Herston, QLD 4006, Australia

## 1. Introduction

Adequate nutrition is essential from the early stages of life onward, to ensure proper growth and development as well as long-term health. Malnutrition, whether undernutrition resulting from macro- and micronutrient deficiencies or overnutrition (obesity) as a result of higher consumption of foods with high macronutrients and low micronutrients, is a global concern, and efforts are needed to prevent it from occurring in early childhood. Such efforts include promoting optimal infant and young child feeding. When feeding practices and nutrition in early life are not optimal, malnutrition and associated poor child health outcomes will arise that lead to morbidity and mortality at a young age, with the effects possibly persisting into adulthood [1,2]. For example, specific dietary patterns can be observed in childhood, persisted over the life course and associated with subclinical cardiovascular events in midlife [1], while obesity in childhood increased the risk of cardiovascular events [2]. Thus, it is important for countries to measure and monitor feeding practices and nutrition of young children in order to gauge risk for poor outcomes and provide prevention strategies to mitigate these outcomes. 

In this Introduction to the Special Issue entitled “Australian Feeding Infant and Toddler Study 2021”, we provide the background to Feeding Infants and Toddlers Studies (FITS) which have, in the past two decades, provided much-needed data on early childhood nutrition that can lead to development of dietary guidelines specific for the first two years of life [3,4]. Appreciating the role FITS can play puts into context the findings of the most recent FITS conducted in Australia, called OzFITS [5,6,7,8,9]. Our purpose is to position OzFITS as a necessary and important step for healthcare professionals in Australia to have for developing dietary guidelines and nutrition education resources for its young children. As will be reported for FITS in the United States, having dietary data and feeding practices for young children can lead to important public health recommendations for a nation.

## 2. Dietary Guidelines and Targets for Young Children 

The World Health Organization (WHO) and UNICEF in 2003 jointly developed the Global Strategy for Infant and Young Child Feeding (IYCF) with the aim of improving nutritional status, growth and development, and health of infants and young children. This Strategy laid the foundation for: raising awareness of problems affecting infant and young child feeding; increasing the commitment of concerned parties including governments toward optimal feeding practices for infants and young children; and providing information for caregivers so they could make informed choices about optimal feeding practices for their children [10]. Since that time, many improvements have been seen yet issues related to IYCF remain in need of attention. 

Thus, to address the need for ongoing monitoring and surveillance of IYCF, in May 2012, a Comprehensive Implementation Plan on Maternal, Infant and Young Child Nutrition was released by WHO. Of the six global targets, three were directed to IYCF: reducing stunting and wasting in children under 5, halting the epidemic of obesity, and increasing the rate of exclusive breastfeeding. The Action Plan included targets to be achieved by 2025 [11]. To allow for more time in achieving the targets, in 2019 an extension was made to have targets achieved by 2030, and to make some targets more stringent. The following are those targets relevant to IYCF:

- Reduction in the number of children under 5 who are stunted;
- Reduce and maintain childhood overweight to less than 3%;
- Increase the rate of exclusive breastfeeding in the first 6 months up to at least 70%;
- Reduce and maintain childhood wasting to less than 3%.

While these initiatives by WHO and UNICEF provide the necessary guidance for countries around the world, for many countries, there are unique circumstances of IYCF that should be addressed by the country itself. Often, this is in the form of Dietary Guidelines that are tailored for the population, taking into account cultural preferences. Yet, many countries have not prioritized ongoing measurement and monitoring of IYCF, especially in the under two years of age (“Under 2”) group of children. In this regard, for the past two decades an international initiative, spearheaded by the Nestle Research Center (Lausanne, Switzerland), has been facilitating IYCF surveys many of which focus on the Under 2 age group [3,4]. 

## 3. Feeding Infants and Children Studies (FITS)

The first FITS began in the United States in 2002 as a large and comprehensive dietary intake study of infants, toddlers and preschoolers aged 24 to 48 months [12]. Since that time, as will be described below, more FITS have been completed that include many countries worldwide [5,13,14,15,16,17,18,19,20,21]. 

### 3.1. Completed FITS Now Include Australia

The FITS studies that have been completed and published are shown in Table 1 [5,13,14,15,16,17,18,19,20,21]. These studies have been ongoing, and since 2002, there have been seven countries having published results, including the OzFITS studies now published in this Special Issue [4,5,6,7,8]. With each survey this is an opportunity to improve or modify the methodology yet stay consistent with a common analysis approach in more recent FITS such as those in Australia, China, Mexico, the Philippines, Russia, and the United States [3,4]. In several countries, FITS have been repeated, which allows for either monitoring of feeding practices over time (i.e., United States) or accessing different age groups (i.e., United States and United Arab Emirates). 

### 3.2. Measurements Used in FITS 

The measurements used in FITS studies have changed over time since 2002, but all have been comprehensive as to dietary assessment and feeding behaviors of Infants and Young Children (IYC). As the most recent FITS, OzFITS provides a detailed description of methodology and illustrates the extent to which dietary and feeding practices data have been collected [5]. In brief, OzFITS gathered dietary intakes using a 24 h recall (repeated in 30% of the group to allow for calculation of usual intakes), while other FITS have used two and sometimes three days of recall intake. Detailed questionnaires and measurements were completed to gather data in the following six modules:

- Caregiver socio-demographics;
- Childbirth details and anthropometric measures;
- Breastfeeding history and use of breastmilk substitutes;
- Timing of introduction of complementary foods and common allergens;
- Use of commercial baby foods and mode of feeding;
- Dietary supplement use.

### 3.3. USA Dietary Guidelines for Children < 24 mo Derived Using US-FITS Results

Three US FITS have been completed since 2002, with cycles 2 and 3 having data on IYC ages 0 to 24 months [14,15]. These studies provided data on feeding practices and dietary intakes of American children < 24 months that had not been collected in previous national surveys. The US FITS (all cycles) has the largest dataset on children’s feeding practices and intakes in the United States during early growth and development [4]. Included are data on intake of breast milk, formula, other beverages, foods, and micronutrient supplements. The results of these FITS were useful in the development of the Dietary Guidelines for Americans released in 2020 which for the first time included recommendations specific to children under 24 months of age. Data from US FITS Cycles 2 and 3 were used by the National Academies of Science Committee on Scoping Existing Guidelines for Feeding Recommendations for IYC Under Age 2 [22]. 

The recommendations for feeding IYC under 2 y are:

- Feed infants human milk for the first 6 months, if possible;
- Provide infants supplemental vitamin D beginning soon after birth;
- Introduce infants to nutrient-dense complementary foods at about 6 months old;
- Introduce infants to potentially allergenic foods along with other complementary foods;
- Encourage infants and toddlers to consume a variety of complementary foods and beverages to meet energy and nutrient needs;
- Establish a healthy beverage pattern.

Note that the recommendations differ from WHO/UNICEF targets as some in the list above are given in the context of caregivers in the United States. As an example, the breastfeeding recommendation is qualified by “if possible” as many working mothers do not have sufficient maternity leave and/or facilities for pumping milk. Vitamin D is recommended as a result of survey data showing poor vitamin D status in ICY living in many parts of the United States. A healthy beverage pattern is recommended as unhealthy sugary beverages have become normalized for children. 

Another application of the US FITS has been the development of a Total Diet Quality Index for Toddlers [23]. This Index is a measurement tool to assess the dietary quality of children between the ages of 1 and 2 years, and uses the United States nutrition guidelines as recommendations to be met. Such tools can give guidance to inform country-based nutrition policies, programs, and practices in order to improve diets of children. Thus, FITS provide country-specific data important for generating public health recommendations for IYCF. 

## 4. The Australian Feeding Infants and Children Study (OzFITS)

As shown in Table 1, OzFITS is the latest FITS, and results are provided in four papers in this Special Issue [5,6,7,8] with a concluding paper [9]. These researchers indicated that national nutrition surveys conducted in Australia over the past 30 years had not included sampling of children under 2 years of age [5]. More than 10 years ago, the 2010 Australian National Infant Feeding Survey captured breastfeeding rates and early caregiver feeding practices but may be too out of date for framing dietary guidelines. Thus, OzFITS is timely. Its design [5] is similar to other FITS conducted elsewhere [3]. The data it provides include the following:

- Breastfeeding and early feeding practices [6];
- Usual nutrient intake distribution and prevalence of inadequacy [7];
- Food intake of Australian toddlers 12–24 months compared to recommendations [8].

The researchers who conducted OzFITS have brought forth recommendations for public health actions based on the data collected and analyzed to date [9]. More analyses may be forthcoming as data are used for secondary analyses and comparative studies.

## 5. Conclusions

As the first two years of life are critical for growth and development, there is a need to invest in nutrition during this critical time to promote optimal growth and development and prevent the occurrence of malnutrition early in life and reduce associated poor health outcomes in later life. Efforts to measure and monitor infant and toddler nutrition have resulted in a growing number of surveys, called Feeding Infants and Toddlers Studies (FITS). In this overview, we have highlight FITS around the world. These surveys can make a substantial contribution to formulating country-specific national dietary guidelines to enhance optimal feeding practices in mothers and/or caregivers for children under 2 years of age. Results from the Australian FITS (OzFITS) are presented in a Special Issue of the journal and is the first survey to provide comprehensive data on nutrition and feeding practices for this age group in Australia, and the latest in a series of FITS studies worldwide. 

## Figures and Tables

**Table 1:** Published Feeding Infants and Toddlers Studies (FITS) by country and date.

FITS by Country	References ^1^	Date of Collection	Sample Size	Age (Months)
United States FIT Cycle 1	[13]	2002	3022	24–48
US FIT Cycle 2	[14]	2008–2009	3273	0–47.9
US FIT Cycle 3	[15]	2015–2016	3235	0–47.9
Philippines	[16]	2013	4218	6–59.9
China	[17]	2011–2012	1409	6–36
Russia	[18]	2013	4612	0–47
Mexico	[19]	2012	2057	0–47
United Arab Emirates −1	[20]	2011–2012	1000	6–24
UAE −2	[21]	2019–2020	525	0–47.9
Australia OzFIT	[5]	2020–2021	1140	0–23.9

^1^ Key reference or reference for design of study.
